# Cancer prevalence in the United States: trends and sociodemographic disparities based on national health interview survey data (2019–2023)

**DOI:** 10.3389/fonc.2026.1790765

**Published:** 2026-07-17

**Authors:** Hafsah Alim Ur Rahman, Afia Salman, Madiha Salman, Rafi Aibani, Syed Ather Hussain, Nausheen Ahmed, Muhammad Sohaib Asghar

**Affiliations:** 1 Department of Internal Medicine, Dow University of Health Sciences, Karachi, Pakistan; 2 Department of Internal Medicine, Charleston Area Medical Center, West Virginia University, Charleston, WV, United States; 3 Department of Medicine, Roswell Park Comprehensive Cancer Center, Buffalo, NY, United States; 4 Division of Hematologic Malignancies and Cellular Therapeutics, Cellular Therapeutics, The University of Kansas Cancer Center, Kansas City, KS, United States; 5 Department of Internal Medicine, AdventHealth Sebring, Sebring, FL, United States

**Keywords:** breast cancer, cancer prevalence, cervical cancer, national health interview survey, prostate cancer, skin cancer

## Abstract

**Introduction:**

Cancer remains a leading cause of mortality in the United States. This study aimed to examine trends and disparities in the prevalence of selected cancers—any cancer, breast, cervical, prostate, and skin cancer—among U.S. adults from 2019 to 2023.

**Materials and methods:**

This cross-sectional study used data from the National Center for Health Statistics (NCHS) Interactive Summary Health Statistics for Adults. Cancer prevalence was based on self-reported physician diagnoses. Joinpoint regression analysis assessed temporal trends, with annual percentage change (APC) estimates and 95% confidence intervals (CI) reported. Analyses were stratified by year, gender, age, race/ethnicity, nativity, veteran status, employment status, geographic region, metropolitan statistical area (MSA), and Social Vulnerability Index (SVI).

**Results:**

The overall prevalence of any cancer remained stable from 2019 (9.6%, 95% CI: 9.3–9.9) to 2023 (9.8%, 95% CI: 9.5–10.1). However, notable disparities were observed. In 2023, White adults had the highest prevalence (11.7%, 95% CI: 11.3–12.2), while Asians had the lowest (3.5%, 95% CI: 2.6–4.8). Breast cancer prevalence in females rose slightly from 3.2% (95% CI: 2.9–3.5) in 2019 to 3.5% (95% CI: 3.2–3.8) in 2023, though not significantly. Cervical cancer in females significantly declined from 1.1% (95% CI: 0.9–1.3) to 0.9% (95% CI: 0.8–1.1) (APC: -6.05, 95% CI: -10.87 to -0.99). Prostate cancer in males rose slightly from 2.3% (95% CI: 2.1–2.6) to 2.5% (95% CI: 2.3–2.8), with Black males having the highest prevalence (3.6%, 95% CI: 2.7–4.7). Skin cancer in females significantly increased from 3.0% (95% CI: 2.7–3.3) to 3.3% (95% CI: 3.0–3.6) (APC: 2.25, 95% CI: 1.11 to 3.36), with the highest prevalence among White adults (4.7%, 95% CI: 4.4–5.0). Higher prevalence was generally observed among older adults, residents of non-MSA areas and the Midwest, individuals with low social vulnerability, the unemployed, U.S.-born individuals, and veterans.

**Conclusion:**

Although overall cancer prevalence remained relatively stable between 2019 and 2023, significant disparities persist across demographic, geographic, and socioeconomic groups. These findings emphasize the need for targeted cancer control strategies to address ongoing inequities.

## 1 Introduction

Cancer constitutes the second most common cause of death in the United States and is the primary cause of death for individuals aged 75 years or younger (1, 2). A population-based cross-sectional study highlighted the impact of the coronavirus disease 2019 (COVID-19) pandemic on disrupting the cancer diagnosis trends, with lower-than-expected cancer incidence recorded in the initial two years (3). Analysis of data obtained from the National Cancer Institute (NCI) Surveillance, National Program of Cancer Registries (NPCR) of the Centers for Disease Control and Prevention (CDC), and the Surveillance, Epidemiology, and End Results (SEER) revealed a decline in the cancer mortality, but a rise in the cancer incidence for women compared to men (2). Nonetheless, the substantial burden of cancer in the U.S. mirrors the demographic transition of baby boomers into the age range with the highest risk of cancer development (4). When comparing with global burden of the disease, 196.5 deaths per 100,000 population are projected to be attributable to any neoplasm, which is slightly higher in United States (312.52 deaths per 100,000 population) (5).

Mortality rates for certain cancers declined in the past years, attributable to the reduction in smoking rates, earlier diagnoses, and improvement in therapeutic options; however, these improvements are threatened by the rise in the incidence of other cancer types despite the progress (6). This reflects the persistent shortcomings in cancer prevention strategies. Notably, the decline in the age-adjusted incidence of cancer is paralleled by an increase in the number of people diagnosed with cancer (7, 8). A report formulated by the American Cancer Society (ACS) further highlighted racial and socioeconomic disparities in the prevalence of risk factors, preventive cancer care, cancer screening, and cancer and palliative treatment (9). Additionally, the increase in cancer survivors has substantiated the cancer prevalence in the U.S.; and is further associated with incremental healthcare expenditures (10). The most common cancers in the U.S. include breast, lung, colorectal, uterine, and prostate cancer (11).

From an epidemiological perspective, understanding the current trends in the prevalence and existing disparities can offer valuable insights into the transforming cancer dynamics in the U.S. This may allow the development of cancer control strategies that are tailored to the gender- and race-specific trends and account for the diverse impact of different cancer types (12). In this study, we aimed to explore the trends and disparities in the prevalence of cancer, including breast cancer, cervical cancer, prostate cancer, and skin cancer, in the U.S. adult population from 2019 to 2023.

## 2 Materials and methods

### 2.1 Study design and data source

This study assessed the prevalence of cancer among U.S. adults aged 18 years and older, focusing on five categories (1): any cancer, (2) breast cancer, (3) cervical cancer, (4) prostate cancer, and (5) skin cancer. Data were obtained from the National Center for Health Statistics (NCHS) Interactive Summary Health Statistics for Adults, conducted by the CDC. The survey utilized a clustered sampling design with home-based interviews to select dwellings, with telephone-only interviews implemented during the COVID-19 pandemic (13). As the dataset was publicly available, Institutional Review Board approval was not required.

### 2.2 Variable definitions and data abstraction

Cancer prevalence was determined through self-reported physician diagnoses. Respondents were asked whether they had ever been informed by a healthcare professional that they had cancer or a malignancy of any kind. Those who answered affirmatively were further asked to specify the type of cancer (14, 15). Breast cancer, cervical cancer, and prostate cancer prevalence were identified based on respondent-reported diagnoses. Individuals who reported a history of cancer were asked to specify the site of malignancy, and those identifying breast, cervical, or prostate cancer were classified accordingly (14, 16–18). Skin cancer prevalence included respondents who self-reported a diagnosis of melanoma, non-melanoma skin cancer, or an unspecified type of skin cancer (14, 19).

Survey data were stratified by year, gender, age, race/ethnicity, nativity, veteran status, employment status, geographic region, metropolitan statistical area (MSA), and Social Vulnerability Index (SVI). The SVI, derived from U.S. Census data, measures community vulnerability based on four domains: socioeconomic status, household composition, race/language status, and housing/transportation. The SVI score ranges from 0 (lowest vulnerability) to 1 (highest vulnerability) and is categorized into four levels: little to no vulnerability (0–0.2500), low vulnerability (0.2501–0.5000), medium vulnerability (0.5001–0.7500), and high vulnerability (0.7501–1.0000) (20). Demographic variables were obtained from the National Health Interview Survey (NHIS) household roster, sample adult, and sample child components (14).

### 2.3 Statistical analysis

Prevalence estimates were reported as percentages with 95% confidence intervals (CI). Joinpoint regression analysis was conducted to assess temporal trends in prevalence, with annual percentage change (APC) estimates and statistical significance determined using 95% CIs. Differences between groups were interpreted based on non-overlapping confidence intervals, which is a standard approach in large population-based datasets. Notably, due to the aggregated nature of publicly available NHIS data, individual-level statistical testing was not feasible.

## 3 Results

### 3.1 Any type of cancer

**Figure 1:**
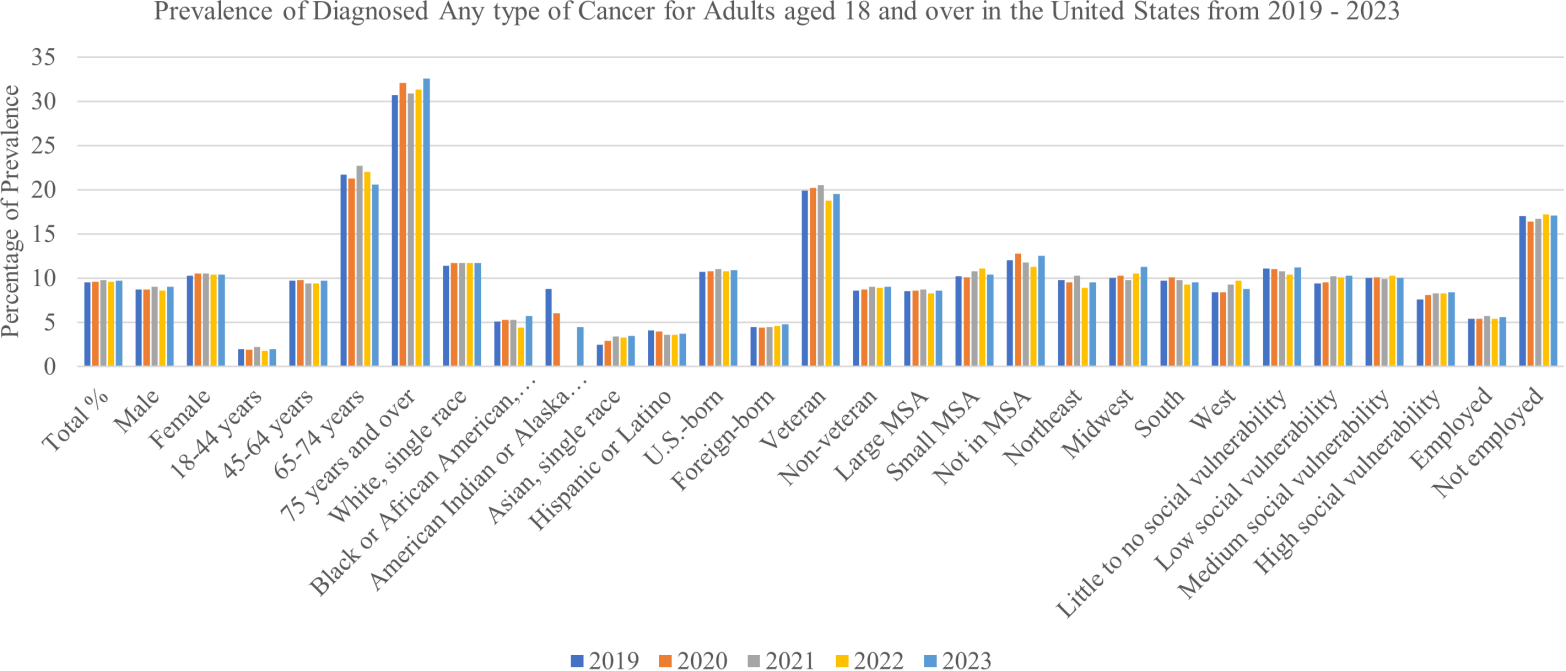
Prevalence of any type of cancer for adults 18 and over in the United States 2019-2023.

**Figure 2:**
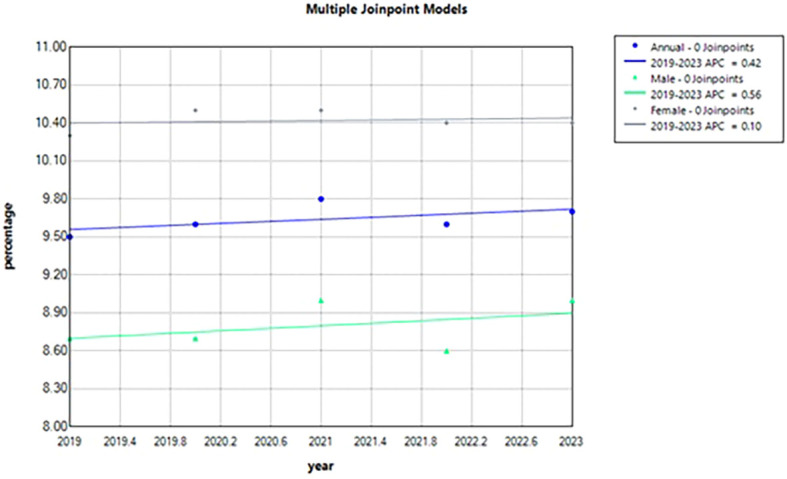
Annual percentage change of any type of cancer for adults 18 and over in the United States 2019-2023.

The prevalence of diagnosed any type of cancer among U.S. adults aged 18 and over remained steady overall from 2019 to 2023, with an insignificant APC of 0.42 (95% CI: -0.46 to 1.28). Females had consistently higher prevalence rates than their male counterparts, which showed no significant change in trends over the study period (APC: 0.56; 95% CI: -1.30 to 2.43). Male prevalence also remained steady over time with an insignificant APC of 0.098 (95% CI: -1.16 to 1.38). Diagnosed cancer was most prevalent in adults aged 75 years or older, with a peak prevalence of 32.6 (95% CI: 30.9 to 34.3) in 2023. **Table 1**; **Figures 1**, **2**; **Supplementary Table 1**.

**Table 1:** Prevalence percentage of cancer in adults aged 18 and over, United States, 2019—2023.

	Any type of cancer					Breast cancer					Cervical cancer					Prostate cancer					Any skin cancer				
Year	2019	2020	2021	2022	2023	2019	2020	2021	2022	2023	2019	2020	2021	2022	2023	2019	2020	2021	2022	2023	2019	2020	2021	2022	2023
Total %	9.5(9.1, 9.9)	9.6(9.3, 10.0)	9.8(9.4, 10.2)	9.6(9.2, 9.9)	9.7(9.3, 10.1)	1.7(1.5, 1.8)	1.7(1.6, 1.9)	1.7(1.6, 1.9)	1.7(1.5, 1.8)	1.8(1.7, 2.0)	1.1(0.9, 1.3)	1(0.8, 1.2)	1(0.8, 1.2)	0.8(0.6, 0.9)	0.9(0.8, 1.1)	2.3(2.1, 2.6)	2.5(2.2, 2.7)	2.4(2.2, 2.7)	2.3(2.0, 2.6)	2.5(2.3, 2.8)	3.1(2.9, 3.3)	3.4(3.2, 3.6)	3.3(3.1, 3.5)	3.3(3.1, 3.6)	3.4(3.2, 3.6)
Male	8.7(8.1, 9.2)	8.7(8.2, 9.2)	9(8.5, 9.5)	8.6(8.1, 9.2)	9(8.5, 9.5)	0(0.0, 0.1)	0(0.0, 0.1)	0(0.0, 0.0)	0(0.0, 0.1)	0.1(0.0, 0.1)	–	–	–	–	–	2.3(2.1, 2.6)	2.5(2.2, 2.7)	2.4(2.2, 2.7)	2.3(2.0, 2.6)	2.5(2.3, 2.8)	3.2(2.9, 3.5)	3.7(3.3, 4.0)	3.4(3.1, 3.8)	3.5(3.2, 3.8)	3.5(3.2, 3.9)
Female	10.3(9.7, 10.8)	10.5(10.0, 11.0)	10.5(10.0, 11.0)	10.4(9.9, 11.0)	10.4(9.9, 10.9)	3.2(2.9, 3.5)	3.3(3.0, 3.6)	3.3(3.1, 3.6)	3.2(2.9, 3.5)	3.5(3.2, 3.8)	1.1(0.9, 1.3)	1(0.8, 1.2)	1(0.8, 1.2)	0.8(0.6, 0.9)	0.9(0.8, 1.1)	–	–	–	–	–	3(2.7, 3.3)	3.1(2.8, 3.4)	3.2(2.9, 3.5)	3.2(2.9, 3.5)	3.3(3.0, 3.6)
18–44 years	2(1.8, 2.4)	1.9(1.7, 2.3)	2.2(1.8, 2.5)	1.8(1.6, 2.1)	2(1.7, 2.3)	0.2(0.1, 0.3)	0.2(0.1, 0.3)	0.2(0.1, 0.3)	0.2(0.1, 0.3)	0.2(0.1, 0.3)	0.8(0.6, 1.1)	0.7(0.4, 1.0)	0.8(0.5, 1.1)	0.5(0.3, 0.7)	0.6(0.4, 0.8)	0(0.0, 0.1)	–	0(0.0, 0.1)	–	0(0.0, 0.1)	0.4(0.3, 0.5)	0.5(0.4, 0.7)	0.5(0.3, 0.6)	0.4(0.3, 0.5)	0.5(0.4, 0.6)
45–64 years	9.7(1.8, 2.4)	9.8(1.7, 2.3)	9.4(1.8, 2.5)	9.4(1.6, 2.1)	9.7(1.7, 2.3)	1.7(1.5, 2.0)	1.8(1.5, 2.1)	1.6(1.4, 1.9)	1.6(1.3, 1.9)	1.8(1.5, 2.2)	1.5(1.1, 1.9)	1.5(1.2, 1.9)	1.4(1.1, 1.8)	0.9(0.6, 1.3)	1.4(1.0, 1.8)	0.9(0.7, 1.2)	1.3(1.0, 1.7)	1.5(1.1, 2.0)	1(0.7, 1.3)	1.2(0.9, 1.6)	3.1(2.8, 3.5)	3.2(2.9, 3.6)	3.1(2.7, 3.5)	3(2.6, 3.4)	3.2(2.8, 3.6)
65–74 years	21.7(20.4, 23.0)	21.3(20.1, 22.7)	22.7(21.4, 24.0)	22(20.8, 23.3)	20.6(19.5, 21.8)	4.1(3.5, 4.7)	3.9(3.4, 4.6)	4.1(3.6, 4.8)	3.5(3.0, 4.1)	3.8(3.3, 4.4)	1.4(0.9, 2.1)	1(0.6, 1.4)	0.8(0.5, 1.4)	1.1(0.7, 1.6)	1.2(0.9, 1.7)	8(6.7, 9.4)	7(5.9, 8.2)	7.1(5.9, 8.5)	7.3(6.2, 8.6)	7.5(6.5, 8.7)	7.5(6.7, 8.4)	8.1(7.3, 9.0)	8(7.2, 8.9)	8.4(7.5, 9.4)	7.6(6.9, 8.5)
75 years and over	30.7(29.0, 32.4)	32.1(30.4, 33.8)	30.9(29.2, 32.7)	31.3(29.7, 33.0)	32.6(30.9, 34.3)	6.1(5.3, 7.0)	6.3(5.4, 7.4)	6.5(5.7, 7.5)	6.8(5.9, 7.8)	6.9(6.0, 7.8)	0.7(0.4, 1.1)	1(0.6, 1.6)	0.6(0.3, 1.0)	1.2(0.7, 1.9)	0.7(0.4, 1.1)	13.6(11.8, 15.5)	15.1(13.2, 17.1)	13.4(11.6, 15.5)	13.2(11.4, 15.2)	14.5(12.6, 16.5)	11.1(10.1, 12.2)	11.9(10.7, 13.1)	11.7(10.6, 12.8)	12.2(11.1, 13.5)	12.5(11.3, 13.7)
White, single race	11.4(10.9, 11.8)	11.7(11.2, 12.2)	11.7(11.2, 12.2)	11.7(11.3, 12.2)	11.7(11.3, 12.2)	1.9(1.7, 2.0)	1.9(1.8, 2.2)	1.9(1.8, 2.1)	1.9(1.8, 2.1)	2(1.8, 2.2)	1.3(1.1, 1.6)	1.2(1.0, 1.5)	1.2(1.0, 1.4)	1(0.8, 1.2)	1.1(0.9, 1.4)	2.6(2.3, 2.9)	2.7(2.5, 3.1)	2.6(2.3, 3.0)	2.6(2.3, 3.0)	2.7(2.4, 3.0)	4.2(3.9, 4.4)	4.6(4.3, 4.9)	4.5(4.2, 4.8)	4.5(4.2, 4.9)	4.7(4.4, 5.0)
Black or African American, single race	5.1(4.4, 6.0)	5.3(4.5, 6.1)	5.3(4.6, 6.2)	4.4(3.7, 5.2)	5.7(4.8, 6.6)	1.1(0.8, 1.6)	1.2(0.9, 1.7)	1.4(1.0, 1.8)	1.3(1.0, 1.8)	1.5(1.1, 2.0)	0.5(0.3, 0.9)	0.6(0.3, 1.1)	0.2(0.0, 0.5)	0.1(0.0, 0.4)	0.6(0.3, 1.1)	2.9(2.1, 3.9)	3.5(2.6, 4.6)	2.9(2.1, 4.0)	2(1.3, 2.9)	3.6(2.7, 4.7)	0.2(0.1, 0.5)	0.1(0.0, 0.4)	0.1(0.0, 0.3)	0.1(0.0, 0.2)	0.2(0.0, 0.4)
American Indian or Alaska Native, single race	8.8(5.1, 14.1)	6(3.2, 10.2)	–	–	4.5(2.4, 7.6)	–	0.6(0.0, 2.7)	1.3(0.2, 4.1)	0.2(0.0, 1.8)	0.6(0.1, 2.4)	–	–	1.2(0.1, 4.9)	1(0.1, 4.1)	0.9(0.1, 3.9)	–	0.4(0.0, 3.8)	–	–	0.7(0.0, 4.4)	0.6(0.1, 2.5)	0.7(0.1, 2.7)	0.8(0.1, 3.0)	-	0.5(0.0, 2.3)
Asian, single race	2.5(1.8, 3.4)	2.9(2.0, 4.0)	3.4(2.6, 4.3)	3.3(2.3, 4.6)	3.5(2.6, 4.8)	1.1(0.6, 1.8)	0.8(0.4, 1.4)	1.7(1.1, 2.4)	1(0.5, 1.7)	1.6(0.9, 2.5)	0.3(0.0, 1.0)	–	0.2(0.0, 0.8)	0(0.0, 0.4)	0.1(0.0, 0.7)	0.8(0.3, 1.7)	0.5(0.1, 1.2)	0.8(0.3, 1.7)	0.3(0.0, 0.9)	0.9(0.3, 2.2)	-	0.3(0.0, 0.8)	0(0.0, 0.3)	0.2(0.1, 0.6)	0(0.0, 0.3)
Hispanic or Latino	4.1(3.3, 5.0)	4(3.3, 4.8)	3.6(3.0, 4.3)	3.6(3.0, 4.2)	3.7(3.1, 4.3)	1(0.7, 1.5)	1.1(0.7, 1.6)	0.6(0.4, 0.9)	0.6(0.4, 0.9)	0.7(0.5, 1.0)	0.8(0.5, 1.2)	0.7(0.4, 1.3)	0.5(0.2, 1.0)	0.5(0.3, 0.9)	0.4(0.2, 0.8)	0.7(0.4, 1.2)	0.6(0.3, 1.1)	1.1(0.6, 1.8)	1.1(0.7, 1.8)	1(0.6, 1.5)	0.5(0.3, 0.8)	0.3(0.2, 0.6)	0.3(0.2, 0.6)	0.2(0.1, 0.4)	0.3(0.2, 0.5)
U.S.-born	10.7(10.2, 11.1)	10.8(10.4, 11.3)	11(10.6, 11.5)	10.8(10.4, 11.3)	10.9(10.5, 11.4)	1.8(1.6, 1.9)	1.8(1.7, 2.0)	1.9(1.7, 2.1)	1.8(1.7, 2.0)	2(1.8, 2.2)	1.2(1.0, 1.4)	1.1(0.9, 1.3)	1.1(0.9, 1.3)	0.9(0.7, 1.1)	1.1(0.9, 1.3)	2.6(2.3, 2.9)	2.8(2.5, 3.1)	2.7(2.4, 3.0)	2.6(2.3, 2.9)	2.8(2.5, 3.1)	3.7(3.5, 4.0)	4(3.8, 4.3)	4(3.7, 4.2)	4(3.8, 4.3)	4.1(3.9, 4.4)
Foreign-born	4.5(3.8, 5.3)	4.4(3.7, 5.2)	4.5(3.9, 5.2)	4.6(3.9, 5.4)	4.8(4.2, 5.5)	1.2(0.8, 1.5)	1.1(0.8, 1.6)	1.1(0.8, 1.5)	0.9(0.7, 1.3)	1.3(1.0, 1.7)	0.7(0.3, 1.3)	0.3(0.1, 0.8)	0.4(0.2, 0.7)	0.3(0.1, 0.5)	0.4(0.1, 0.7)	1.1(0.7, 1.7)	1.3(0.9, 1.9)	1.4(0.8, 2.1)	1.3(0.8, 1.9)	1.2(0.8, 1.8)	0.6(0.4, 0.9)	0.7(0.5, 1.0)	0.7(0.5, 1.0)	0.7(0.4, 1.0)	0.6(0.4, 0.9)
Veteran	19.9(18.3, 21.6)	20.2(18.6, 21.8)	20.5(18.8, 22.4)	18.8(17.2, 20.6)	19.5(17.8, 21.2)	0.5(0.3, 0.9)	0.6(0.2, 1.4)	0.3(0.1, 0.6)	0.5(0.2, 1.1)	0.3(0.2, 0.7)	–	2.6(0.9, 5.9)	–	–	0.7(0.1, 2.5)	7.3(6.3, 8.4)	6.6(5.7, 7.7)	7.5(6.3, 8.7)	6.1(5.1, 7.2)	7.4(6.3, 8.6)	7.9(6.9, 9.1)	8.5(7.5, 9.7)	8.4(7.3, 9.6)	7.8(6.7, 9.0)	7.7(6.6, 9.0)
Non-veteran	8.6(8.2, 9.0)	8.7(8.3, 9.1)	9(8.6, 9.4)	8.9(8.6, 9.3)	9(8.6, 9.4)	1.8(1.6, 1.9)	1.8(1.6, 2.0)	1.9(1.7, 2.0)	1.8(1.6, 1.9)	2(1.8, 2.1)	1.1(0.9, 1.3)	0.9(0.8, 1.1)	0.9(0.8, 1.1)	0.8(0.6, 1.0)	0.9(0.8, 1.1)	1.5(1.2, 1.7)	1.7(1.5, 2.0)	1.7(1.4, 1.9)	1.7(1.5, 2.0)	1.7(1.5, 2.0)	2.7(2.5, 2.9)	2.9(2.7, 3.2)	3(2.7, 3.2)	3.1(2.8, 3.3)	3.1(2.9, 3.3)
Large MSA	8.5(8.0, 9.0)	8.6(8.1, 9.1)	8.7(8.2, 9.2)	8.3(7.9, 8.8)	8.6(8.2, 9.1)	1.6(1.4, 1.8)	1.7(1.5, 1.9)	1.7(1.5, 1.9)	1.5(1.3, 1.7)	1.7(1.5, 1.9)	1(0.8, 1.2)	0.7(0.5, 0.9)	0.7(0.5, 0.9)	0.6(0.5, 0.9)	0.5(0.4, 0.7)	2.2(1.9, 2.6)	2.2(1.9, 2.5)	2.5(2.1, 2.9)	2.2(1.8, 2.5)	2.2(1.9, 2.5)	2.7(2.4, 2.9)	3(2.7, 3.2)	2.8(2.5, 3.1)	2.7(2.4, 3.0)	2.9(2.6, 3.2)
Small MSA	10.2(9.4, 10.9)	10.1(9.5, 10.8)	10.8(10.1, 11.6)	11.1(10.4, 11.9)	10.4(9.7, 11.0)	1.6(1.4, 1.9)	1.7(1.4, 2.0)	1.7(1.4, 2.0)	1.8(1.5, 2.1)	1.9(1.6, 2.1)	1.4(1.0, 1.7)	1.4(1.0, 1.8)	1.3(1.0, 1.7)	0.8(0.6, 1.2)	1.2(0.9, 1.6)	2.4(1.9, 2.9)	2.7(2.2, 3.2)	2.5(2.1, 3.0)	2.4(1.9, 2.9)	2.8(2.3, 3.3)	3.6(3.2, 4.0)	3.6(3.2, 4.0)	4.1(3.7, 4.5)	4.3(3.8, 4.8)	3.8(3.4, 4.3)
Not in MSA	12(10.9, 13.1)	12.8(11.6, 14.0)	11.8(10.4, 13.2)	11.3(10.2, 12.4)	12.5(11.5, 13.5)	2(1.6, 2.5)	1.9(1.5, 2.3)	2(1.6, 2.5)	1.9(1.5, 2.3)	2.4(1.9, 3.0)	1.1(0.6, 1.9)	1.4(0.8, 2.2)	1.3(0.8, 2.0)	1.1(0.8, 1.7)	2(1.4, 2.8)	2.5(1.9, 3.3)	3.2(2.4, 4.0)	2.2(1.6, 3.0)	2.6(1.9, 3.3)	3.4(2.7, 4.3)	3.8(3.3, 4.4)	4.5(3.7, 5.3)	3.7(3.1, 4.4)	3.9(3.3, 4.6)	4.5(3.9, 5.2)
Northeast	9.8(8.8, 10.9)	9.5(8.7, 10.4)	10.3(9.4, 11.3)	8.9(8.0, 9.8)	9.5(8.5, 10.5)	1.8(1.5, 2.3)	2.1(1.7, 2.5)	2.2(1.8, 2.7)	1.7(1.3, 2.1)	2.1(1.7, 2.6)	1(0.6, 1.5)	0.6(0.3, 0.9)	0.5(0.3, 0.9)	0.3(0.1, 0.6)	0.5(0.2, 0.8)	2.7(2.1, 3.4)	2.8(2.2, 3.5)	2.6(1.9, 3.4)	2(1.4, 2.7)	2.3(1.7, 3.0)	2.9(2.4, 3.3)	2.7(2.3, 3.1)	3.3(2.8, 3.9)	2.5(2.1, 3.0)	2.9(2.4, 3.4)
Midwest	10(9.2, 10.8)	10.3(9.5, 11.2)	9.8(9.0, 10.7)	10.5(9.7, 11.3)	11.3(10.5, 12.1)	1.6(1.3, 1.9)	1.7(1.3, 2.1)	1.7(1.4, 2.0)	1.9(1.5, 2.2)	1.9(1.6, 2.2)	1.3(0.9, 1.7)	1.3(0.8, 1.9)	1.1(0.7, 1.7)	0.8(0.5, 1.2)	1.4(1.0, 1.9)	2.4(1.9, 2.9)	2.9(2.3, 3.6)	2.6(2.0, 3.2)	2.1(1.6, 2.7)	3.1(2.5, 3.8)	3.2(2.8, 3.7)	3.7(3.2, 4.2)	3.1(2.7, 3.5)	3.6(3.1, 4.1)	4.3(3.8, 4.8)
South	9.7(9.2, 10.3)	10.1(9.4, 10.7)	9.8(9.2, 10.5)	9.3(8.7, 9.9)	9.5(8.9, 10.0)	1.7(1.5, 2.0)	1.7(1.4, 2.0)	1.6(1.4, 1.9)	1.5(1.2, 1.7)	1.7(1.5, 2.0)	1.1(0.9, 1.5)	1.2(0.9, 1.6)	1(0.7, 1.3)	0.8(0.5, 1.1)	0.9(0.7, 1.2)	2.6(2.1, 3.1)	2.6(2.2, 3.1)	2.6(2.1, 3.1)	2.3(1.9, 2.8)	2.4(2.0, 2.9)	3.3(3.0, 3.7)	3.7(3.3, 4.1)	3.4(3.0, 3.7)	3.4(3.1, 3.8)	3.4(3.1, 3.8)
West	8.4(7.6, 9.2)	8.4(7.7, 9.2)	9.3(8.5, 10.1)	9.7(9.0, 10.4)	8.8(8.1, 9.6)	1.5(1.3, 1.9)	1.6(1.3, 1.9)	1.6(1.3, 1.9)	1.8(1.5, 2.1)	1.7(1.4, 2.1)	1.1(0.7, 1.5)	0.7(0.5, 1.1)	1.1(0.8, 1.5)	1.1(0.7, 1.6)	0.9(0.6, 1.3)	1.5(1.2, 2.0)	1.6(1.2, 2.1)	2(1.6, 2.6)	2.6(2.1, 3.3)	2.4(2.0, 3.0)	2.9(2.5, 3.3)	3.1(2.7, 3.6)	3.4(2.9, 3.8)	3.6(3.1, 4.1)	3(2.5, 3.4)
Little to no social vulnerability	11.1(10.2, 12.0)	11(10.1, 12.0)	10.8(9.9, 11.9)	10.4(9.5, 11.4)	11.2(10.2, 12.3)	1.7(1.4, 2.1)	2(1.5, 2.5)	1.7(1.4, 2.1)	1.7(1.3, 2.2)	1.9(1.5, 2.4)	1.1(0.7, 1.6)	1(0.6, 1.5)	0.9(0.6, 1.4)	0.6(0.3, 1.1)	1.3(0.8, 2.0)	2.7(2.1, 3.4)	2.8(2.3, 3.5)	2.5(1.9, 3.1)	2.7(2.0, 3.6)	2.7(2.0, 3.5)	3.9(3.4, 4.5)	4.1(3.6, 4.6)	4.3(3.8, 4.9)	4(3.3, 4.7)	4.3(3.7, 5.1)
Low social vulnerability	9.4(8.8, 10.1)	9.5(8.9, 10.2)	10.2(9.5, 10.9)	10.1(9.3, 10.8)	10.3(9.6, 11.1)	1.7(1.4, 2.0)	1.6(1.3, 1.9)	1.9(1.6, 2.2)	1.6(1.3, 1.9)	1.9(1.6, 2.2)	1.4(1.0, 1.9)	1(0.7, 1.3)	1.2(0.8, 1.6)	0.8(0.6, 1.2)	0.8(0.5, 1.1)	2.4(1.9, 2.9)	2.5(2.0, 3.0)	2.3(1.9, 2.9)	2.2(1.7, 2.7)	2.9(2.3, 3.5)	3.1(2.8, 3.5)	3.5(3.1, 4.0)	3.7(3.3, 4.1)	3.6(3.1, 4.1)	4(3.5, 4.5)
Medium social vulnerability	10(9.3, 10.8)	10.1(9.4, 10.8)	9.9(9.2, 10.6)	10.3(9.6, 11.1)	10(9.3, 10.7)	1.8(1.5, 2.1)	1.9(1.6, 2.2)	1.8(1.5, 2.1)	1.8(1.5, 2.2)	2(1.7, 2.3)	1.1(0.8, 1.5)	1.2(0.9, 1.6)	0.9(0.6, 1.3)	0.8(0.5, 1.1)	0.8(0.6, 1.2)	2.2(1.8, 2.7)	2.4(2.0, 2.9)	2.9(2.4, 3.4)	2.3(1.8, 2.8)	2.5(2.0, 3.0)	3.3(2.9, 3.8)	3.3(2.9, 3.7)	3.2(2.8, 3.6)	3.7(3.3, 4.2)	3.6(3.2, 4.1)
High social vulnerability	7.6(6.9, 8.4)	8.1(7.3, 8.9)	8.3(7.6, 9.1)	8.3(7.7, 8.9)	8.4(7.8, 9.0)	1.5(1.2, 1.9)	1.5(1.2, 1.8)	1.5(1.2, 1.9)	1.6(1.3, 1.8)	1.6(1.3, 1.9)	0.8(0.6, 1.2)	0.8(0.4, 1.3)	0.8(0.6, 1.2)	0.8(0.5, 1.1)	0.9(0.7, 1.3)	2(1.6, 2.6)	2.3(1.7, 2.9)	2(1.5, 2.6)	2.2(1.8, 2.7)	2.3(1.9, 2.8)	2.2(1.8, 2.5)	2.7(2.3, 3.2)	2.2(1.8, 2.6)	2.6(2.2, 2.9)	2.4(2.1, 2.8)
Employed	5.4(5.1, 5.8)	5.4(5.1, 5.8)	5.7(5.4, 6.1)	5.4(5.0, 5.8)	5.6(5.3, 6.0)	0.8(0.7, 1.0)	0.8(0.7, 1.0)	0.8(0.7, 1.0)	0.7(0.6, 0.9)	0.9(0.7, 1.0)	1(0.8, 1.3)	1(0.7, 1.2)	1(0.8, 1.3)	0.6(0.4, 0.8)	0.8(0.6, 1.0)	0.8(0.7, 1.0)	1(0.8, 1.2)	1(0.8, 1.3)	0.8(0.6, 1.0)	0.9(0.7, 1.1)	1.9(1.7, 2.1)	2(1.8, 2.2)	1.9(1.7, 2.1)	1.9(1.7, 2.1)	2.1(1.9, 2.3)
Not employed	17(16.2, 17.8)	16.4(15.6, 17.2)	16.7(15.9, 17.5)	17.2(16.4, 17.9)	17.1(16.3, 17.8)	3.2(2.9, 3.6)	3.1(2.8, 3.5)	3.2(2.9, 3.6)	3.3(3.0, 3.6)	3.6(3.2, 4.0)	1.2(0.9, 1.6)	1(0.8, 1.3)	0.9(0.7, 1.2)	1.1(0.8, 1.4)	1.1(0.9, 1.4)	5.9(5.2, 6.6)	5.7(5.0, 6.4)	5.6(4.9, 6.3)	5.9(5.2, 6.6)	6.1(5.5, 6.9)	5.5(5.1, 5.9)	5.7(5.2, 6.1)	5.8(5.4, 6.3)	6.1(5.6, 6.6)	5.9(5.5, 6.4)

CDC, Centers for Disease Control; MSA, Metropolitan Statistical Area.

When analyzing racial data, White adults had the highest prevalence rates, with a peak prevalence of 11.7 (95% CI: 11.3 to 12.2) from 2020 to 2023. American Indian or Alaska Natives had the second highest rates, which fell from 8.8 (95% CI: 5.1 to 14.1) in 2019 to 4.5(95% CI: 2.4 to 7.6) in 2023. Black or African American adults saw a peak prevalence of 5.7(95% CI: 4.8 to 6.6) in 2023, while Hispanics or Latinos reported a decline in prevalence rates from 4.1 (95% CI: 3.3 to 5.0) in 2019 to 3.7(95% CI: 3.1 to 4.3) in 2023. Asians had the lowest cancer prevalence rates, with 3.5 (95% CI: 2.6 to 4.8) as the highest in 2023.

Non-MSA regions had the highest prevalence with a peak of 12.5 (95% CI: 11.5 to 13.5) in 2023. Adults living in small MSA areas had a prevalence of 10.2 (95% CI: 9.4 to 10.9) in 2019, which rose to 11.1 (95% CI: 10.4 to 11.9) in 2022 before falling to 10.4 (95% CI: 9.7 to 11.0) in 2023. The large MSA population showed the lowest prevalence, with a peak prevalence of 8.7 (95% CI: 8.2 to 9.2) in 2021 and remained relatively steady by the end of the study period. In census regions, the Midwest had the highest prevalence, while the West had the lowest. Midwestern rates increased from 10 (95% CI: 9.2 to 10.8) in 2019 to 11.3 (95% CI: 10.5 to 12.1) in 2023 while in the West, rates first rose from 8.4 (95% CI: 7.6 to 9.2) in 2019 to 9.7 (95% CI: 9.0 to 10.4) in 2022 and then fell again to 8.8 (95% CI: 8.1 to 9.6) in 2023.

Little to no social vulnerability group had consistently higher prevalence rates than other social vulnerability groups, with a peak prevalence of 11.2 (95% CI: 10.2 to 12.3) in 2023. For the employed population, the prevalence of diagnosed cancer remained relatively stable over the study period. It started at 5.4 (95% CI: 5.1 to 5.8) in 2019 and showed a slight increase to 5.6 (95% CI: 5.3 to 6.0) in 2023. In contrast, the unemployed population experienced significantly higher prevalence rates compared to their employed counterparts. The prevalence in this group was 17 (95% CI: 16.2 to 17.8) in 2019 and remained high, reaching 17.1 (95% CI: 16.3 to 17.8) in 2023. U.S.-born individuals had consistently higher prevalence rates compared to foreign-born individuals, with U.S.-born prevalence increasing from 10.7 (95% CI: 10.2 to 11.1) in 2019 to 10.9 (95% CI: 10.5 to 11.4) in 2023, while foreign-born prevalence rose from 4.5 (95% CI: 3.8 to 5.3) in 2019 to 4.8 (95% CI: 4.2 to 5.5) in 2023. Veterans had higher prevalence rates compared to non-veterans, with veteran prevalence decreasing from 19.9 (95% CI: 18.3 to 21.6) in 2019 to 19.5 (95% CI: 17.8 to 21.2) in 2023, while non-veteran prevalence rose from 8.6 (95% CI: 8.2 to 9.0) in 2019 to 9.0 (95% CI: 8.6 to 9.4) in 2023.

### 3.2 Breast cancer

**Figure 3:**
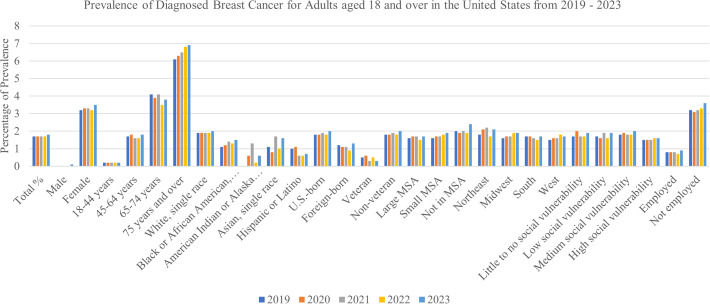
Prevalence of diagnosed breast cancer for adults 18 and over in the United States 2019-2023.

**Figure 4:**
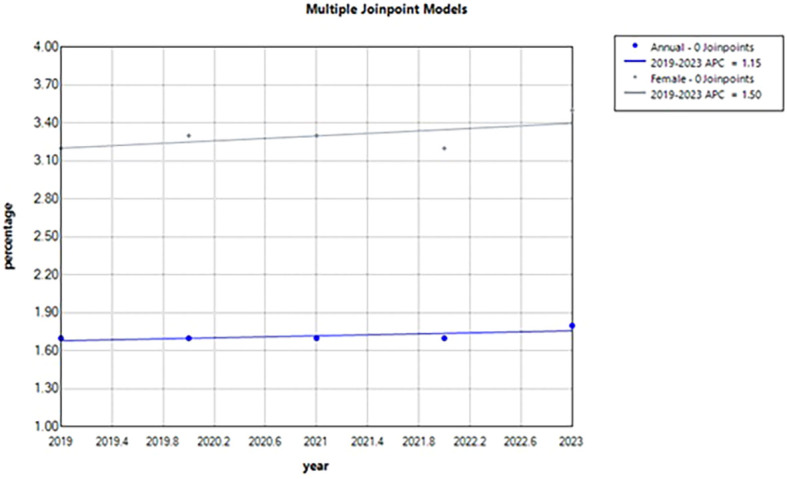
Annual percentage change of breast cancer for adults 18 and over in the United States 2019-2023.

Breast cancer prevalence remained steady, with the highest rate in 2023 of 1.8 (95% CI: 1.7 to 2.0) (APC: 1.1497; 95% CI: -0.7739 to 3.0743). Breast cancer prevalence in males was negligible, while the prevalence in females insignificantly increased from 3.2 (95% CI: 2.9 to 3.5) in 2019 to 3.5 (95% CI: 3.2 to 3.8) in 2023 (APC 1.50; 95% CI: -1.0193 to 4.0572). The highest prevalence was found in adults aged 75 years or older, which hit a peak in 2023 of 6.9 (95% CI: 6.0 to 7.8). **Table 1**; **Figures 3**, **4**; **Supplementary Table 1**.

Racially, White adults had the highest prevalence, which remained relatively steady and reached 2.0 (95% CI: 1.8 to 2.2) in 2023. The Asian population hit a peak of 1.7 (95% CI: 1.1 to 2.4) in 2021 and then slightly decreased to 1.6 (95% CI: 0.9 to 2.5) in 2023. Prevalence rates in Black or African Americans hit a peak of 1.5 (95% CI: 1.1 to 2.0) in 2023. American Indians or Alaska Natives had the lowest prevalence, their highest being 1.3 (95% CI: 0.2 to 4.1) in 2021.

Population residing in non-MSA areas had the highest prevalence, peaking at 2.4 (95% CI: 1.9 to 3.0) in 2023. In contrast, large MSA areas had the lowest prevalence, reaching 1.7 (95% CI: 1.5 to 1.9) as its highest in 2023. The Northeast and the Midwest census regions showed the highest prevalence, while the South and West had similar rates that weren’t far behind. Regardless, rates either increased or stayed stable in all four regions.

Little to no social vulnerability group had consistently higher prevalence rates than other social vulnerability groups, with a peak prevalence of 1.9 (95% CI: 1.5 to 2.4) in 2023. For the employed population, the prevalence of diagnosed breast cancer remained relatively stable over the study period. It started at 0.8 (95% CI: 0.7 to 1.0) in 2019 and showed a slight increase to 0.9 (95% CI: 0.7 to 1.0) in 2023. In contrast, the unemployed population experienced higher prevalence rates compared to their employed counterparts. The prevalence in this group was 3.2 (95% CI: 2.9 to 3.6) in 2019 and remained high, reaching 3.6 (95% CI: 3.2 to 4.0) in 2023. U.S.-born individuals had consistently higher prevalence rates compared to foreign-born individuals, with U.S.-born prevalence increasing from 1.8 (95% CI: 1.6 to 1.9) in 2019 to 2 (95% CI: 1.8 to 2.2) in 2023, while foreign-born prevalence slightly rose from 1.2 (95% CI: 0.8 to 1.5) in 2019 to 1.3 (95% CI: 1.0 to 1.7) in 2023. Veterans had lower prevalence rates compared to non-veterans, with veteran prevalence decreasing from 0.5 (95% CI: 0.3 to 0.9) in 2019 to 0.3 (95% CI: 0.2 to 0.7) in 2023, while non-veteran prevalence rose from 1.8 (95% CI: 1.6 to 1.9) in 2019 to 2 (95% CI: 1.8 to 2.1) in 2023.

### 3.3 Cervical cancer

**Figure 5:**
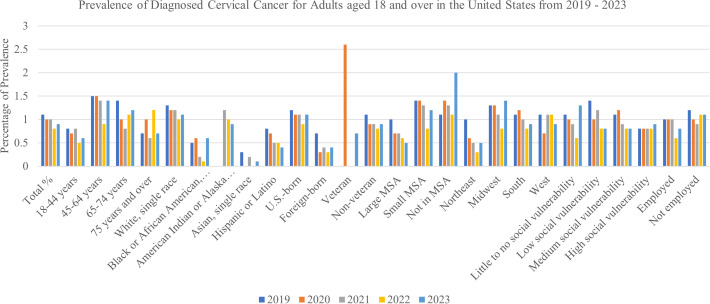
Prevalence of diagnosed cervical cancer for adults 18 and over in the United States 2019-2023.

**Figure 6:**
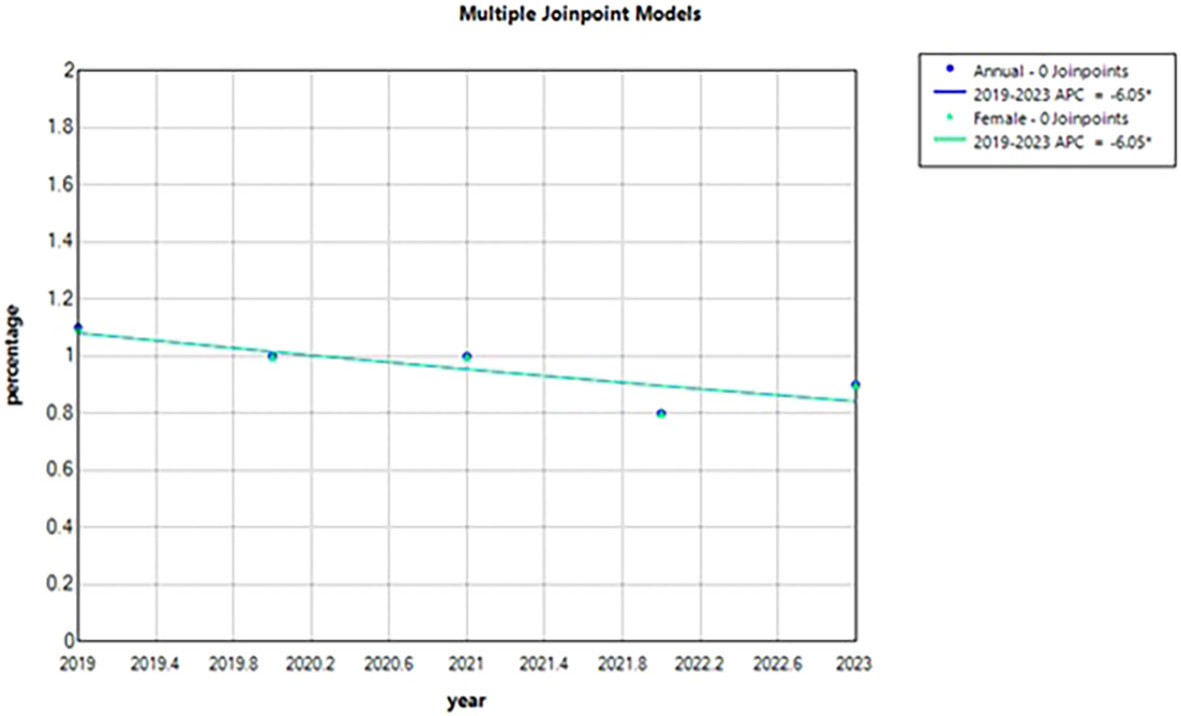
Annual percentage change of cervical cancer for adults 18 and over in the United States 2019-2023.

The prevalence of diagnosed cervical cancer among U.S. females aged 18 and over significantly decreased from 1.1 (95% CI: 0.9 to 1.3) in 2019 to 0.9 (95% CI: 0.8 to 1.1) in 2023 with an APC of -6.05* (95% CI: -10.87 to -0.99). Diagnosed cervical cancer was most prevalent in women aged 45 to 64 years, with a peak prevalence of 1.5 (95% CI: 1.2 to 1.9) in the first two years, after which it decreased slightly. **Table 1**, **Figures 5**, **6**, **Supplementary Table 1**.

When analyzing racial data, White adults had the highest prevalence rates, with a peak prevalence of 1.3 (95% CI: 1.1 to 1.4) in 2023. The American Indian or Alaska Native population had the second highest prevalence, with a maximum of 1.2 (95% CI: 0.1 to 4.9) in 2021.

Black or African American adults saw a peak prevalence of 0.6 (95% CI: 0.3 to 1.1) in 2023. Hispanic or Latino adults reported a prevalence of 0.8 (95% CI: 0.5 to 1.2) in 2019, which fell to 0.4 (95% CI: 0.2 to 0.8) in 2023. Black or African Americans and Asians had the lowest prevalence rates.

Non-MSA regions had the highest prevalence with a peak of 2.0 (95% CI: 1.4 to 2.8) in 2023, while the Large MSA population showed the lowest prevalence rates with 0.5 (95% CI: 0.4 to 0.7) in 2023. In census regions, the Midwest had the highest prevalence, while the Northeast had the lowest. Prevalence decreased in all regions from 2019 to 2023, except the Midwest, where it slightly rose from 1.3 (95% CI: 0.9 to 1.7) in 2019 to 1.4 (95% CI: 1.0 to 1.9) in 2023.

Little to no social vulnerability group faced the steepest increase in prevalence from 1.1 (95% CI: 0.7 to 1.6) in 2019 to 1.3 (95% CI: 0.8 to 2.0) in 2023. The low social vulnerability group reported the steepest decrease in rates from 1.4 (95% CI: 1.0 to 1.9) in 2019 to 0.8 (95% CI: 0.5 to 1.1) in 2023. For the employed population, the prevalence started at 1.0 (95% CI: 0.8 to 1.3) in 2019 and showed a decrease to 0.8 (95% CI: 0.6 to 1.0) in 2023. In contrast, the unemployed population experienced higher prevalence rates compared to their employed counterparts. The prevalence in this group was 1.2 (95% CI: 0.9 to 1.6) in 2019 and slightly fell to 1.1 (95% CI: 0.9 to 1.4) in 2023. U.S.-born individuals had consistently higher prevalence rates compared to foreign-born individuals, with U.S.-born prevalence decreasing from 1.2 (95% CI: 1.0 to 1.4) in 2019 to 1.1 (95% CI: 0.9 to 1.3) in 2023. The prevalence in foreign-born individuals had a steeper decrease from 0.7 (95% CI: 0.3 to 1.3) in 2019 to 0.4 (95% CI: 0.1 to 0.7) in 2023. Prevalence decreased in both veterans and non-veterans, but the former saw a steeper decrease from 2.6 (95% CI: 0.9 to 5.9) in 2020 to 0.7 (95% CI: 0.1 to 2.5) in 2023.

### 3.4 Prostate cancer

**Figure 7:**
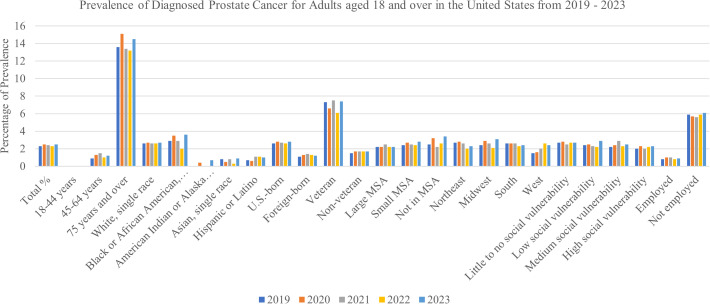
Prevalence of diagnosed prostate cancer for adults 18 and over in the United States 2019-2023.

**Figure 8:**
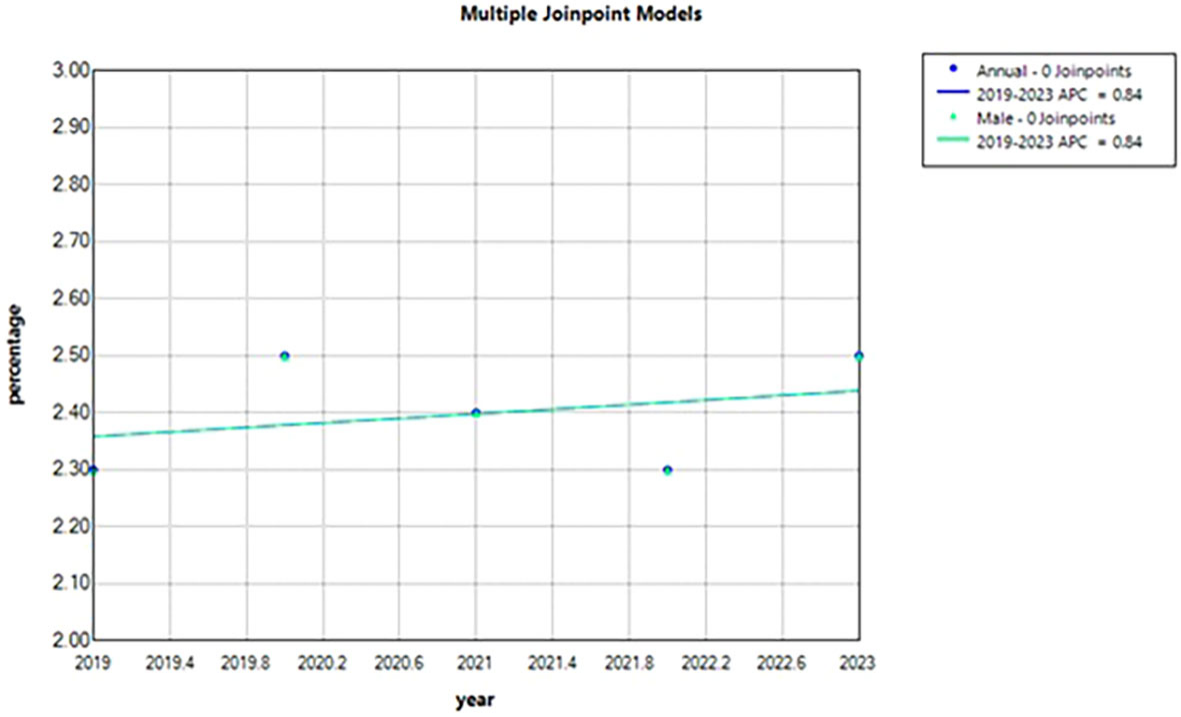
Annual percentage change of prostate cancer for adults 18 and over in the United States 2019-2023.

The prevalence of prostate cancer in males in the U.S. aged 18 or over increased slightly from 2.3 (95% CI: 2.1 to 2.6) in 2019 to 2.5 (95% CI: 2.3 to 2.8) in 2023 with an insignificant APC of 0.84 (95% CI: -3.43 to 5.2). Diagnosed prostate cancer was consistently higher in prevalence in adults aged 75 years or older, with the rates settling at 14.5 (95% CI: 12.6 to 16.5) in 2023. **Table 1**; **Figures 7**, **8**; **Supplementary Table 1**.

The Black or African American population had the highest prostate cancer prevalence, reaching a peak of 3.6 (95% CI: 2.7 to 4.7) in 2023. White adults had the next highest numbers and remained relatively stable during the study period, with a prevalence rate of 2.7 (95% CI: 2.4 to 3.0) at the end of the study period. Asians had the lowest prevalence, with a peak of 0.9 (95% CI: 0.3 to 2.2) in 2023.

Non-MSA areas had the highest prevalence, rising from 2.5 (95% CI: 1.9 to 3.3) in 2019 to 3.4(2.7 to 4.3) in 2023. Large MSA regions have the lowest prevalence, with a peak rate of 2.5 (95% CI: 2.1 to 2.9) in 2021, after which they fell slightly. The Midwest region had the highest prevalence, rising from 2.4 (95% CI: 1.9 to 2.9) in 2019 to 3.1 (95% CI: 2.5 to 3.8) in 2023. Northeastern and Southern prevalence wasn’t far behind, but decreased overall during the study period. West had the lowest rates but increased from 1.5 (95% CI: 1.2 to 2.0) in 2019 to 2.4 (95% CI: 2.0 to 3.0) in 2024.

Little to no social vulnerability group had higher prevalence rates than other social vulnerability groups, with peak prevalence of 2.7 (95% CI: 2.0 to 3.5) in 2023. Even though the low social vulnerability group had lower rates from 2019 to 2022, but then increased steeply to 2.9 (95% CI: 2.3 to 3.5) in 2023. The high social vulnerability group had the lowest prevalence. For the employed population, the prevalence of diagnosed prostate cancer remained relatively stable over the study period. It started at 0.8 (95% CI: 0.7 to 1.0) in 2019 and showed a slight increase to 0.9 (95% CI: 0.7 to 1.0) in 2023. In contrast, the unemployed population experienced higher prevalence rates, which were 5.9 (95% CI: 5.2 to 6.6) in 2019 and remained high, reaching 6.1 (95% CI: 5.5 to 6.9) in 2023. U.S.-born individuals had consistently higher prevalence rates compared to foreign-born individuals, with U.S.-born prevalence increasing from 2.6 (95% CI: 2.3 to 2.9) in 2019 to 2.8 (95% CI: 2.5 to 3.1) in 2023. Veterans had higher prevalence rates compared to non-veterans, with veteran prevalence decreasing from 7.3 (95% CI: 6.3 to 8.4) in 2019 to 7.4 (95% CI: 6.3 to 8.6) in 2023, while non-veteran prevalence rose from 1.5 (95% CI: 1.2 to 1.7) in 2019 to 1.7 (95% CI: 1.5 to 2.0) in 2023.

### 3.5 Any skin cancer

**Figure 9:**
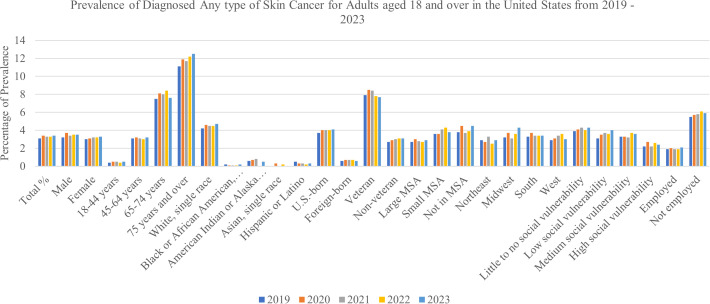
Prevalence of diagnosed any type of skin cancer for adults 18 and over in the United States 2019-2023.

**Figure 10:**
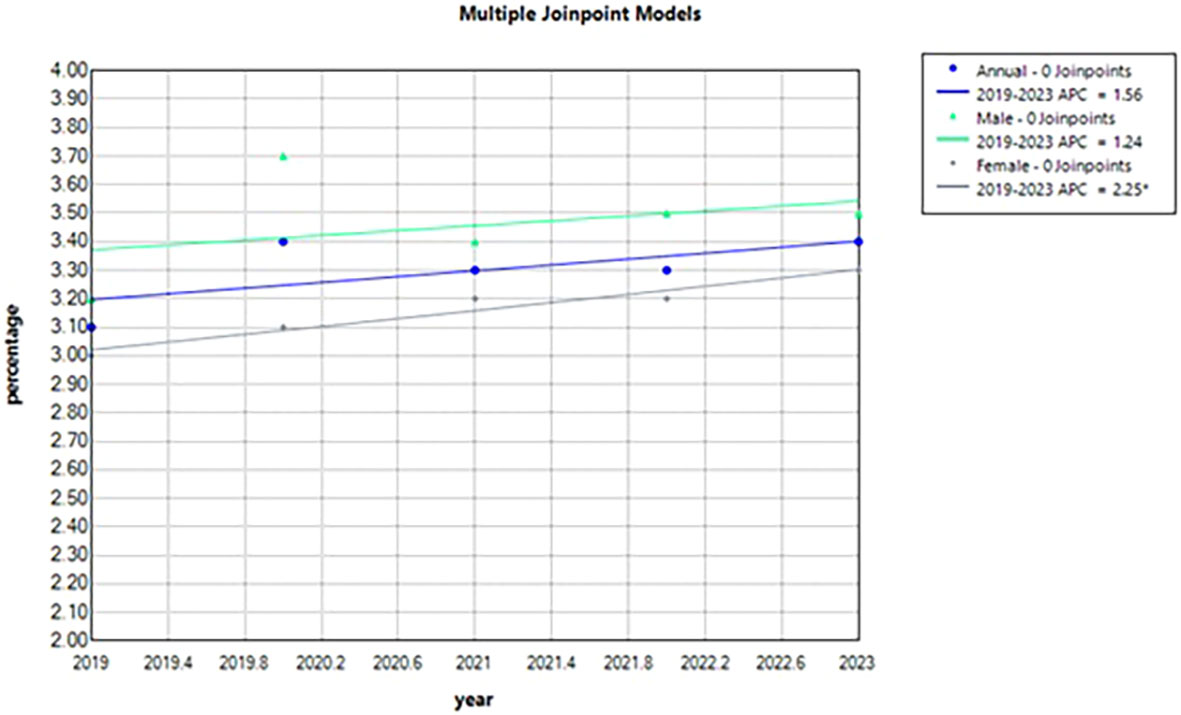
Annual percentage change of any type of skin cancer for adults 18 and over in the United States 2019-2023.

Overall diagnosed skin cancer prevalence in adults in the U.S. increased from 3.1 (95% CI: 2.9 to 3.3) in 2019 to 3.4 (95% CI: 3.2 to 3.6) in 2023 with an insignificant APC of 1.56 (95% CI: -1.01 to 4.10). Males had consistently higher rates compared to females, with prevalence rising from 3.2 (95% CI: 2.9 to 3.5) in 2019 to 3.5 (95% CI: 3.2 to 3.9) in 2023 (APC: 1.24; 95% CI: -2.15 to 4.69). Female prevalence significantly increased from 3.0 (95% CI: 2.7 to 3.3) in 2019 to 3.3 (95% CI: 3.0 to 3.6) in 2023 (APC: 2.25*; 95% CI: 1.11 to 3.36). Diagnosed skin cancer was most prevalent in adults aged 75 years or older, with a peak prevalence of 12.5 (95% CI: 11.3 to 13.7) in 2023. **Table 1**; **Figures 9**, **10**; **Supplementary Table 1**.

White adults had the highest prevalence rates, with a peak prevalence of 4.7 (95% CI: 4.4 to 5.0) in 2023. American Indian or Alaska Native rose from 0.6 (95% CI: 0.1 to 2.5) in 2019 to 0.8 (95% CI: 0.1 to 3.0) in 2021 but then fell again to 0.5 (95% CI: 0.0 to 2.3) in 2023. Prevalence in the Hispanic or Latino race also decreased from 0.5 (95% CI: 0.3 to 0.8) in 2019 to 0.3 (95% CI: 0.2 to 0.5) in 2023. Black or African American adults and Asians had the lowest prevalence.

Non-MSA regions had the highest prevalence with a peak of 4.5 (95% CI: 3.9 to 5.2) in 2023. The large MSA population showed the lowest prevalence, with its highest of 3.0 (95% CI: 2.7 to 3.2) in 2020. In census regions, the Midwest had the highest prevalence, while the Northeast had the lowest. Midwestern rates increased from 3.2 (95% CI: 2.8 to 3.7) in 2019 to 4.3 (95% CI: 3.8 to 4.8) in 2023. Overall, rates increased in all four regions from 2019 to 2023.

Little to no social vulnerability group had consistently higher prevalence rates than other social vulnerability groups, with a peak prevalence of 4.3 (95% CI: 3.7 to 5.1) in 2023. For the employed population, the prevalence of diagnosed skin cancer remained relatively stable over the study period. It started at 1.9 (95% CI: 1.7 to 2.1) in 2019 and showed a slight increase to 2.1 (95% CI: 1.9 to 2.3) in 2023. In contrast, the unemployed population experienced higher prevalence rates. The prevalence in this group was 5.5 (95% CI: 5.1 to 5.9) in 2019 and increased to 5.9 (95% CI: 5.5 to 6.4) in 2023. U.S.-born individuals had consistently higher prevalence rates compared to foreign-born individuals, with U.S.-born prevalence increasing from 3.7% (95% CI: 3.5 to 4.0) in 2019 to 4.1% (95% CI: 3.9 to 4.4) in 2023. Veterans had higher prevalence rates compared to non-veterans, with veteran prevalence decreasing from 7.9 (95% CI: 6.9 to 9.1) in 2019 to 7.7 (95% CI: 6.6 to 9.0) in 2023, while non-veteran prevalence rose from 2.7 (95% CI: 2.5 to 2.9) in 2019 to 3.1 (95% CI: 2.9 to 3.3) in 2023.

## 4 Discussion

This study reports the recent disparities and trends in the prevalence of cancer types 2019–2023 among the general U.S. adult population. The study findings indicated that the cancer prevalence among U.S. adults remained stable overall across most cancer types, with minor fluctuations in the prevalence rates. While the overall cancer prevalence demonstrated no significant change over the study period, the prevalence rates remained highest in females, White populations, and in individuals aged 75 years or older, with notable regional and racial disparities. Non-MSA areas and the Midwest region consistently had the highest cancer prevalence. Moreover, breast cancer prevalence remained steady among the U.S. adults, peaking in older women, and higher prevalence rates were observed for White populations and non-MSA residents. Cervical cancer prevalence notably declined, especially among Hispanics and foreign-born women, whereas prostate cancer prevalence increased insignificantly from 2019 to 2023, with the highest prevalence in Black or African American men. Skin cancer prevalence increased over the study period, with prevalence rates peaking in males and White adults. Across all cancer types, unemployed, U.S.-born individuals, and veteran populations generally reported the highest cancer prevalence rates. The results of this study reinforce previous research findings, suggesting a plateau in the incidence and prevalence, especially for common cancers (21).

The statistics report on breast cancer during 2012–2021 by the ACS demonstrated a continuous upward trend in the incidence of breast cancer; however, the overall death rate from breast cancer exhibited a consistent decline during 1989–2022. The statistics report also highlighted a steeper increase in the breast cancer incidence for younger and Asian American/Pacific Islander women (22). Our study demonstrated that the prevalence of breast cancer among the Asian population peaked in 2021 and declined through 2023. Not only are White populations predominantly represented in the datasets, including the SEER program cancer registry (23), but reproductive factors also contribute to the higher incidence of estrogen receptor-positive tumors in White women compared to Black women (24). These explanations may conform to the highest prevalence of breast cancer reported in White populations, as reported in our study. Moreover, compared to non-Hispanic women, women from minority racial/ethnic groups demonstrate a lower incidence of breast cancer at older ages (25). Our study demonstrated higher prevalence rates of breast cancer in women with little to no social vulnerability. Higher breast cancer incidence has been found to be associated with lower social vulnerability and higher mammography screening rates in the U.S (26). While women from rural or non-metropolitan areas have been documented to be diagnosed at later stages of breast cancer, the age-adjusted incidence rates of breast cancer remain high for non-Hispanic White women from urban areas (27).

Based on our study findings, the prevalence of cervical cancer decreased over the study period, 2019–2023. The analysis of SEER data for 2000–2019, however, demonstrated a rise in the incidence of cervical cancer, especially in the low-income counties, despite the initiation of higher sensitivity screening tests (28). On the contrary, the United States Cancer Statistics data analysis during 2007–2020 demonstrated a decline in the cervical cancer incidence rates for women aged 15–29, which may partially be explained by vaccination against human papillomavirus (HPV) and screening for cervical cancer (29). In addition to these factors, the decline in the prevalence of cervical cancer during 2019–2023 can be linked to the drop in cervical cancer screening rates during the COVID-19 pandemic, caused by the closure of screening sites and suspension of screening services (30, 31). Withal, racial and ethnic disparities in the incidence of cervical cancer in the U.S. have been documented in the existing literature. Our study demonstrated the highest prevalence rates in White populations. Based on histological subtypes, the incidence rates were highest in Hispanic and White women for cervical adenocarcinoma, compared to the highest incidence rates of cervical squamous cell carcinoma in Black and Hispanic women (32). This may be explained by variations in the distribution of HPV genotypes and variants (32, 33).

Prostate cancer prevalence trends reported in this study corroborate with the trends reported in the literature. Prostate cancer incidence trends demonstrated a significant decline during 2000–2013, followed by a subsequent increase in the incidence during 2014–2020. The increasing trend is most likely attributed to the United States Preventive Services Task Force (USPSTF) guideline (34). Moreover, studies have consistently demonstrated significant racial disparities in the incidence of prostate cancer, with higher rates in Black men (34, 35). Compared to White men with prostate cancer, Black men with prostate cancer are more likely to have genomic instability, metabolic dysregulation, and elevated androgen receptor signaling (36). Corresponding to the findings of this study, rural Black populations have higher incidence rates of prostate cancer (37). Research suggests a positive association between the incidence rates of prostate cancer and socioeconomic status, and the low prevalence rates of prostate cancer among foreign-born individuals recorded in our study may be explained by the low prostate cancer screening rates and environmental exposure variants compared to U.S.-born individuals (38).

The increasing trend in the prevalence of skin cancer among U.S. adults during 2019–2023 substantiates the incidence and prevalence trends for melanoma, basal cell carcinoma (BCC), and squamous cell carcinoma (SCC) in the U.S. during 1990–2019, with the skin cancer burden remaining stable throughout this period (39). The rise in the incidence rates has been recorded beyond the borders as well, and the factors contributing to the rising incidence include exposure to ultraviolet radiation, air pollution, and depletion of the stratospheric ozone layer (40). Hormonal and immunological differences may account for the higher risk of melanoma among men (41). The incidence of non-melanoma skin cancers is also higher in men compared to women (42). Notably, socially vulnerable populations are more likely to present at later stages and have limited access to healthcare services (43). An increase in the diagnostic scrutiny, such as screening for skin cancer, can be attributed to the increasing incidence trends (44). The higher prevalence rates among the U.S.-born individuals compared to those who were born in foreign countries can be traced to a lower likelihood of sunburns, a lower number of total body examinations, and wearing long-sleeved clothing (45). In addition to this, greater adverse reactions to the sun among non-Hispanic White individuals and the highest incidence of invasive cutaneous melanoma have been observed in these individuals compared to non-Hispanic American Indian/Alaska Native people (46).

Nonetheless, this study has several limitations that shall be acknowledged for a better interpretation of the results. The use of self-reported physician diagnoses may introduce recall bias or misclassification, which could lead to either underestimation or overestimation of true cancer prevalence. The retrospective design further restricts causal inferences and limits the ability to assess individual disease progression over time. Moreover, the analysis does not consider differences in healthcare access, health-seeking behaviors, or regional and population-based variations in diagnostic criteria, all of which may influence the disparities observed. The limited number of time points (2019-2023) in joinpoint analysis are acknowledged to be more cautious when interpreting APC estimates. Lastly, this study does not explore the intersectionality of racial, geographic, and age-related disparities in the prevalence of breast, cervical, prostate, and skin cancer. Future research should aim to identify risk factors and predictors of disparities in the prevalence of these cancer types in the U.S. It is also important for future studies to assess the effectiveness of targeted interventions aimed at reducing the burden of cancer, particularly among high-risk populations.

## 5 Conclusion

In conclusion, this study provides the first comprehensive analysis of disparities and trends in cancer prevalence among U.S. adults from 2019 to 2023. While the overall prevalence of cancer in the U.S. adult population remained largely stable between 2019 and 2023, significant disparities persist across various demographic groups. Females, White individuals, and those aged 75 and older consistently exhibited the highest cancer prevalence rates. Geographically, non-MSA and the Midwest region showed the highest burden of cancer. These findings showing higher prevalence in White, low social vulnerability, and non-MSA populations are likely influenced by differences in screening, access to care, and survivorship rather than underlying risk. These findings underscore the need for targeted public health strategies to address persistent disparities and support high-risk groups effectively. Policy efforts should focus on improving access to early cancer detection and screening services, particularly in underserved regions and among vulnerable populations.

## Data Availability

The original contributions presented in the study are included in the article/**Supplementary Material**. Further inquiries can be directed to the corresponding author.
