# Supplementary material for: Cancer prevalence in the United States: trends and sociodemographic disparities based on national health interview survey data (2019–2023)
Source: Front Oncol. 2026 Jul 17;16:1790765. doi: 10.3389/fonc.2026.1790765 (PMC13423679; doi:10.3389/fonc.2026.1790765)
Supplement: Supplementary file 1 [file Table1.docx]

**Trends and Disparities in the Prevalence of Cancer among US Adults from 2019 to 2023**

| **Cancer Type** | **Variable (year 2019-2023)** | **APC (95% CI)** | **Global projected DALYs per 100,000 population up to the year 2050** | **United States projected DALYs per 100,000 population (year 2050)** |
| --- | --- | --- | --- | --- |
| **Any type of Cancer** | Annual | 0.4176 (-0.4638 to 1.2764) | 4136.18 (3567.75 to 4777.65) | 5726.88 (5070.12 to 6392.99) |
|  | Male | 0.5640 (-1.3045 to 2.4318) | 4524.54 (3889.87 to 5207.33) | 6109.82 (5495.40 to 6756.57) |
|  | Female | 0.0976 (-1.1571 to 1.3804) | 3747.01 (3226.32 to 4374.45) | 5353.85 (4656.98 to 6021.80) |
| **Breast Cancer** | Annual | 1.1497 (-0.7739 to 3.0743) | 344.95 (265.73 to 436.54) | 471.37 (404.50 to 534.20) |
|  | Female | 1.4956 (-1.0193 to 4.0572) | 675.09 (512.97 to 858.93) | 917.70 (786.26 to 1042.12) |
| **Cervical Cancer** | Annual Female | -6.0539* (-10.8730 to -0.9866) | 265.42 (215.13 to 321.67) | 102.94 (90.14 to 117.39) |
| **Prostate Cancer** | Annual Male | 0.8373 (-3.4280 to 5.1903) | 390.78 (306.55 to 495.99) | 799.02 (682.02 to 923.64) |
| **Any Skin Cancer** | Annual | 1.5610 (-1.0107 to 4.0994) | Melanoma: 26.93 (22.62 to 30.54) | Melanoma: 104.85 (94.49 to 115.29) |
|  |  |  | Non-melanoma: 28.18 (22.72 to 33.98) | Non-melanoma: 75.18 (62.72 to 89.76) |
|  | Male | 1.2442 (-2.1472 to 4.6915) | Melanoma: 32.31 (26.79 to 37.67) | Melanoma: 146.04 (129.65 to 162.53) |
|  |  |  | Non-melanoma: 33.38 (27.28 to 40.89) | Non-melanoma: 105.05 (88.65 to 123.52) |
|  | Female | 2.2486* (1.1126 to 3.3599) | Melanoma: 21.53 (16.82 to 25.38) | Melanoma: 64.73 (58.11 to 71.22) |
|  |  |  | Non-melanoma: 22.97 (17.01 to 28.94) | Non-melanoma: 46.07 (37.05 to 56.61) |

**Supplementary Table 1:** Annual percent change (APC) for cancer in adults 18 and above between 2019 to 2023; and global projections for the year 2050 from global burden of disease database.

APC = annual percent change; CI = Confidence Interval; DALYs: Disability adjusted life years.

* Indicates that the annual percentage change (APC) is significantly different from zero at α = 0.05.

Global data extracted from <https://vizhub.healthdata.org/gbd-foresight/>
